# HPVPool-Seq: a genotype-guided pooling strategy for cost-effective next-generation sequencing detection of HPV integration in cervical samples

**DOI:** 10.1128/spectrum.01399-25

**Published:** 2025-08-19

**Authors:** Wenyan Guan, Siyuan Liu, Yiqiang Chen, Chengzhuo Chu, Guanghao Peng, Jinhuan Wang, Qiao Weng, Yali Fu, Jingjing Li

**Affiliations:** 1 Department of Pathology, Nanjing Drum Tower Hospital, The Affiliated Hospital of Nanjing University Medical School 66506, Nanjing, Jiangsu, China; 2 Department of Precision Medical Center, Nanjing Drum Tower Hospital, The Affiliated Hospital of Nanjing University Medical School 66506, Nanjing, Jiangsu, China; 3 Department of Pathology, Women's Hospital of Nanjing Medical University, Nanjing Maternity and Child Health Care Hospital 159379, Nanjing, Jiangsu, China; 4 Department of Precision Medical Center, Nanjing Normal University Nanjing Drum Tower Hospital 66506, Nanjing, Jiangsu, China; 5 Department of Obstetrics and Gynecology, Nanjing Drum Tower Hospital, The Affiliated Hospital of Nanjing University Medical School 66506, Nanjing, Jiangsu, China; 6 Jiangsu Health Development Research Center 154520 , Nanjing, Jiangsu, China; 7 National Health Commission Key Laboratory of Contraceptives Vigilance and Fertility Surveillance, Nanjing, Jiangsu, China; 8 Jiangsu Provincial Medical Key Laboratory of Fertility Protection and Health Technology Assessment, Nanjing, Jiangsu, China; Central Texas Veterans Health Care System, Temple, Texas, USA

**Keywords:** HPV integration, pooling strategy, next-generation sequencing, cost-effectiveness, viral genomics, cervical cancer screening

## Abstract

Integration of high-risk human papillomavirus (hrHPV) DNA is a critical event in carcinogenesis and a promising biomarker for risk stratification. However, the high cost of next-generation sequencing (NGS) limits its widespread clinical adoption. We developed HPVPool-Seq, an innovative pooling strategy that leverages the inherent diversity of HPV genotypes as natural barcodes, enabling cost-effective, scalable integration detection. Samples were pooled based on qPCR-derived HPV genotypes and viral loads prior to targeted NGS and bioinformatic decoding. A web-based automation tool was implemented to streamline pooling and decoding workflows. In a proof-of-concept study of 175 clinical specimens, HPVPool-Seq achieved 77.1% exact genotype concordance and 97.1% combined sensitivity compared with qPCR. Cost simulations demonstrated a 60% reduction in per-sample sequencing expenses. Self-correcting capability through targeted retesting further enhanced reliability. HPVPool-Seq offers a novel, traceable, and economically viable solution for high-throughput HPV integration profiling, balancing cost, scalability, and clinical precision. This strategy sets a new framework for molecular screening in HPV-associated cancers.

**IMPORTANCE:**

Accurate detection of high-risk HPV integration is critical for identifying individuals at true risk of progression to malignancy. However, the high cost of next-generation sequencing (NGS) has limited its widespread clinical application. Here, we propose HPVPool-Seq, a novel pooling-based sequencing strategy that uses HPV genotypes as intrinsic barcodes to guide sample pooling without compromising detection sensitivity. This method dramatically reduces sequencing costs while maintaining genotype-level traceability and offers a built-in mechanism for selective retesting of discordant cases. By addressing both technical and economic barriers, our approach provides a scalable, clinically applicable solution for HPV integration profiling in large cohorts, with important implications for precision screening, triage, and epidemiological surveillance.

## INTRODUCTION

Persistent infection with high-risk human papillomavirus (hrHPV) is a well-established etiological factor in the development of cervical cancer and head-neck cancers (1–3). The integration of HPV DNA into the host genome marks a pivotal transition from transient infection to malignant transformation, correlating with disease progression and therapeutic resistance (4–6). Unlike transient HPV infections that commonly regress, integration events are considered hallmarks of transforming infections and are closely correlated with genomic instability and carcinogenesis (7–9). Current clinical guidelines increasingly emphasize the need for biomarkers to stratify HPV-positive patients into distinct risk categories, where integration status serves as a prognostic indicator for targeted management (10). Therefore, identifying HPV integration status may serve as a potential triage tool to distinguish truly high-risk individuals among HPV-positive women and reduce unnecessary diagnostic procedures and overtreatment. Several studies have identified integration-related biomarkers with clinical implications, including disruption of the E2 gene (11), increased expression of viral–host fusion transcripts, and recurrent integration near oncogenes, such as MYC and FHIT (12, 13). These features have been associated with disease progression, genomic instability, and poor clinical outcomes, highlighting the relevance of integration profiling in HPV-positive individuals. However, existing integration assays remain limited by technical complexity, locus specificity, or low throughput, restricting their utility in large-scale screening and risk stratification.

Traditionally, HPV integration has been assessed using methods such as detection of integrated papillomavirus sequences by ligation-mediated PCR (DIPS-PCR) and amplification of papillomavirus oncogene transcripts (APOT) (14), which are labor-intensive, locus-specific, and not readily scalable for clinical use. With the advent of high-throughput next-generation sequencing (NGS), it has become feasible to perform comprehensive analysis of viral integration at a genome-wide scale (15). Targeted capture sequencing using HPV whole-genome probes enables the enrichment of HPV sequences and detection of integration breakpoints throughout the human genome. This approach allows not only precise genotyping of HPV but also systematic mapping of integration events, offering a more complete understanding of HPV-host interactions (16, 17). However, the high per-sample cost of NGS remains a critical limitation for its application in population-level HPV screening or large-scale epidemiological studies.

To address this challenge, we devised a novel and cost-effective pooling strategy that leverages the inherent biological identity of HPV genotypes as sample-specific barcodes. Since HPV-positive status is routinely determined by PCR-based genotyping assays prior to any downstream analysis, we utilized the HPV genotype and viral load (as estimated by Ct value) to guide the pooling of samples. Samples infected with distinct HPV genotypes were combined into pools, balancing input DNA and estimated viral copy number across pools to ensure comparable sequencing representation. By doing so, each sequencing library represents multiple individual samples, reducing per-sample costs while preserving genotype-level traceability.

In this study, we established and validated this pooling-based sequencing approach for HPV integration detection using a cohort of clinically annotated HPV-positive specimens (175 samples). Our results demonstrate robust concordance between pooled NGS and individual qPCR genotyping, with only less than 3% samples lost in the pooling sequencing, and >75% samples showed exactly genotype matching. Our findings support the feasibility of this strategy for large-scale integration profiling and its potential role in improving the cost-effectiveness of HPV-related cancer prevention programs, supporting its potential translation into routine HPV-positive patient stratification workflows.

## MATERIALS AND METHODS

### Study design

**Fig 1:**
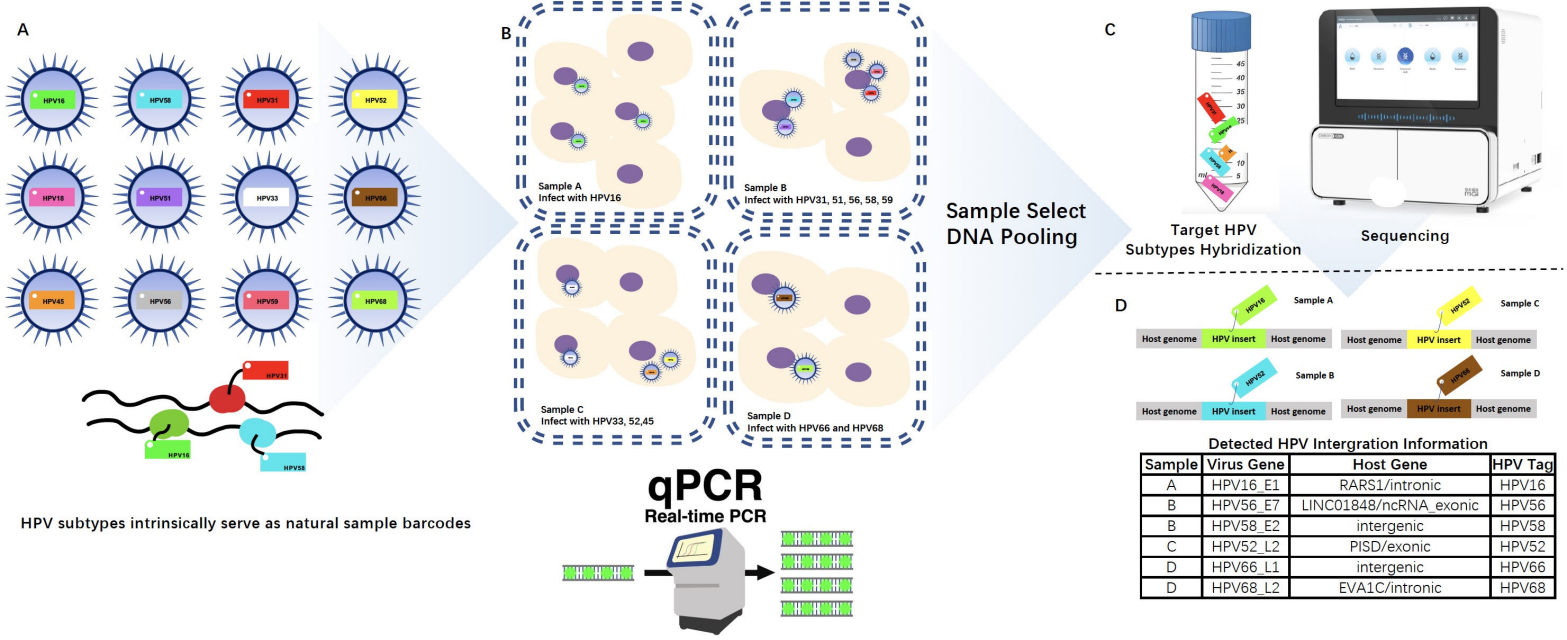
Overview of the HPVPool-Seq strategy. (**A**) Schematic diagram illustrating the pooling strategy design. Samples confirmed as HPV-positive by qPCR were grouped into distinct pools according to genotype and viral load (Ct value). Each pool included 2–5 samples with minimal overlap of HPV genotypes to ensure unique tagging. (**B**) Automated pooling strategy using an R-based script that takes HPV genotype and Ct values as input to generate pool assignments with roughly equalized viral copy number across pools. (**C**) Library construction and sequencing of pooled samples. Viral genome capture was performed using hybridization probes targeting the full HPV genome, followed by high-throughput next-generation sequencing. (**D**) Bioinformatic decoding of sequencing results. HPV genotypes detected in each pool were matched against prior qPCR genotyping to trace back integration signals to individual samples.

This study aims to develop and validate a novel, cost-efficient pooling-based next-generation sequencing (NGS) approach for comprehensive detection of HPV integration events. The core concept leverages the intrinsic specificity of HPV genotypes as biological barcodes (Fig. 1A), allowing for sample pooling without molecular indexing. We first confirmed HPV positivity and determined genotypes through fluorescence quantitative PCR (q-PCR) (Fig. 1B), which is routinely conducted prior to any downstream integration analysis. Based on the HPV genotype and viral load (Ct value), samples with different genotypes were grouped into pools to balance the sequencing depth and minimize cross-sample interference (Fig. 1C). After target capture and whole-genome sequencing of HPV, a computational decoding algorithm was applied to assign integration events back to individual samples using genotype traces and alignment scores (Fig. 1D).

### Samples

Women undergoing cervical screening (either cytology-based or HPV testing) at Nanjing Drum Tower Hospital and Nanjing Maternity and Child Health Hospital from July 2022 to July 2024 were prospectively enrolled. Residual cervical exfoliated cells from HPV-positive specimens were collected following routine cytology (ThinPrep cytologic test, TCT) or hrHPV testing. All included specimens tested positive for one or more of the 14 hrHPV types. Informed consent was obtained, and the study was approved by the institutional ethics committee.

### DNA extraction and quantitative PCR for HPV typing and viral load estimation

DNA was extracted from cervical exfoliated cells using TIANamp Genomic DNA Kit (TIANGEN Biotech, Beijing, China). HPV genotyping and quantification were performed using a fluorescent quantitative PCR (q-PCR) assay based on TaqMan probe technology (HPV DNA Detection Kit, Hongwei Biotechnology, Jiangsu, China), covering 14 hrHPV genotypes: 16, 18, 31, 33, 35, 39, 45, 51, 52, 56, 58, 59, 66, and 68. Amplification and result interpretation followed the manufacturer’s instructions. A Ct value ≤38 with a characteristic S-shaped amplification curve was considered positive. A mixed plasmid standard harboring 14 hrHPV types was used as a reference, allowing rough calibration of Ct values to viral copy number.

Viral copy numbers were estimated from Ct values using the formula Copy = 1/2^Ct^, assuming 100% PCR amplification efficiency. This provides a relative comparison rather than absolute quantification. Logarithmic transformation was applied to improve normality for statistical analysis.

### Sample pooling strategy and library preparation

To reduce per-sample library construction cost, we developed an HPV genotype-guided pooling strategy. Samples were grouped based on distinct HPV genotypes determined by qPCR. Pools were composed of samples infected with non-overlapping HPV genotypes. The pooling volume was adjusted according to Ct values to ensure equal representation of HPV viral load in each pool. Each pool was treated as a single sample for library preparation using standard Illumina-compatible protocols.

### Targeted HPV capture and next-generation sequencing

The HPV probes used for hybrid capture enrichment were designed by MyGenostics (Baltimore, MD, USA) based on the full-length genomes of 32 HPV types. Specifically, the probe utilized is ViralCap_HPV Probe (Cat. No. MS7-M352-24) (18). The pooled libraries underwent hybridization capture followed by sequencing on an Illumina platform (Illumina Inc., San Diego, CA, USA) with paired-end reads. The enriched libraries were sequenced on an Illumina platform (NovaSeq 6000) using 150 bp paired-end reads (PE150), providing sufficient coverage and resolution for integration site mapping.

### Sequencing data processing and HPV integration analysis

Bioinformatics analysis was performed using the human genome hg38 database and 514 reference genomes of 32 HPV types. First, data preprocessing was conducted to remove adapter sequences, low-quality reads, and short sequences (<40 bp). Preprocessed data were then aligned to all HPV reference genomes using BWA (19). Data with an average sequencing depth >10 and >45% coverage at ≥4× depth were filtered to obtain HPV subtyping information. Based on subtyping results, the optimal HPV reference genome was selected, and BWA was used to align data to both hg38 and the selected reference genome to generate BAM files. Structural variants were analyzed using SVdetect to identify preliminary integration site regions, while CREST (20) was used to validate integration sites—CREST results were defined as high-confidence, and results undetected by SVdetect were classified as low confidence. Finally, consensus sequences near integration sites were extracted and collated.

### Development of a web-based analysis tool for HPV pooling strategy

**Fig 2:**
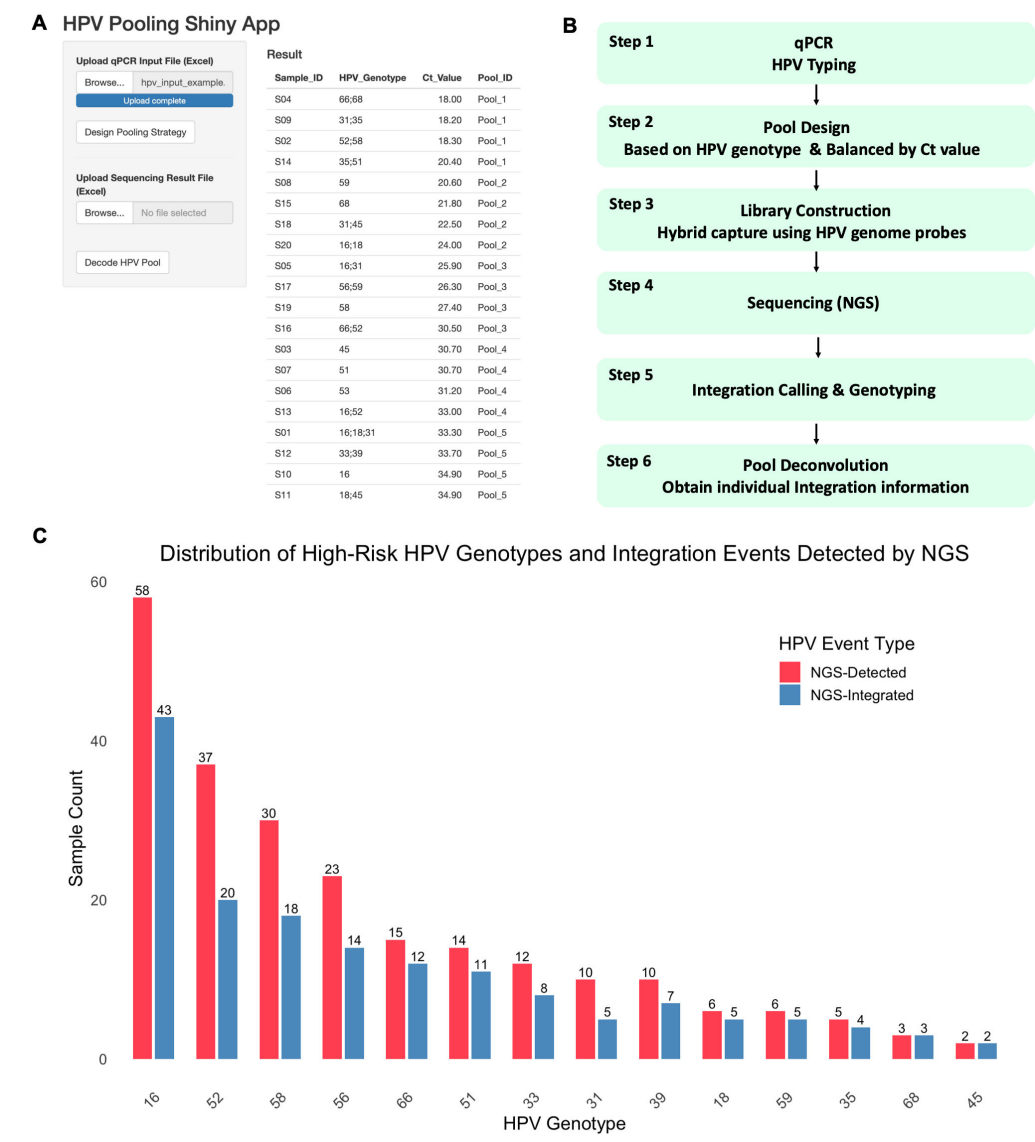
HPVPool-Seq pipeline and performance in clinical samples. (**A**) Custom-built HPVPool-Seq Shiny App interface, supporting two key modules: Design Pooling Strategy based on qPCR genotypes and Ct values, and Decode HPV Pool using sequencing data for genotype deconvolution. (**B**) Schematic workflow of the HPVPool-Seq method. (**C**) Distribution of hrHPV genotypes detected by NGS. Dual bars indicate the total number of samples in which each genotype was detected (NGS-Detected, pink) and the number in which the genotype was found to be integrated into the host genome (NGS-Integrated, blue). Only the 14 clinically recognized hrHPV types were considered.

To facilitate the application and reproducibility of the pooling-based sequencing method, we developed a user-friendly Shiny App using R (v4.3.1). The app contains two core functional modules: automated pooling design and sample decoding. Users are required to upload two Excel files: one containing qPCR-derived HPV genotype and Ct values (for pooling design), and the other containing HPV genotyping results from sequencing with pool assignment (for decoding) (Fig. 2A).

The pooling module leverages HPV genotypes as natural biological barcodes. Samples with distinct HPV genotypes are grouped into pools based on their genotype and estimated viral load (Ct value), ensuring balanced input per pool. The decoding module then reversely matches sequencing-detected HPV genotypes back to the original sample IDs within each pool. The tool was implemented in R using shiny, dplyr, and readxl packages and includes error-handling, interactive data upload, and result export functionalities. The HPV Pooling Shiny App has been deployed online and can be directly accessed at https://li-lab-samllapp.shinyapps.io/hpv_pooling_shiny_app/. If you need the full source code, which includes an R script file featuring two core functional modules, automated pooling design and sample decoding, as well as the demo files, please contact the corresponding author.

### Statistics

To evaluate the concordance between the pooling-based NGS assay and the qPCR-based genotyping reference, we performed a categorical agreement analysis focusing specifically on the 14 hrHPV genotypes. This restriction was based on two rationales: first, the qPCR assay used in this study was designed to detect only 14 clinically relevant hrHPV genotypes (HPV16, 18, 31, 33, 35, 39, 45, 51, 52, 56, 58, 59, 66, and 68); second, only integration events involving high-risk genotypes have established significance for cervical carcinogenesis and clinical triage.

For each sample, the detected hrHPV genotypes from the NGS-pooling method were compared with those obtained by qPCR (gold standard). A match code variable was defined to categorize the comparison results into four classes: Exact Match, all NGS-detected genotypes were identical to qPCR (both in content and count); Partial Miss: NGS missed one or more of the qPCR-detected genotypes but no extra types were detected; Complete Miss: NGS failed to detect any of the types present in qPCR (including undetectable samples); Over-detection: NGS detected additional genotypes not present in qPCR.

## RESULTS

### Development of the HPVPool-Seq workflow

To address the high cost of individual HPV integration detection by next-generation sequencing (NGS), we developed a novel pooling-based method named HPVPool-Seq (HPV genotype-guided pooling sequencing strategy). This strategy utilizes genotype and viral load (Ct value) derived from routine qPCR-based HPV screening as a biological barcode to guide the pooling of samples prior to library construction and target capture sequencing.

As illustrated in Fig. 1, the workflow includes (i) qPCR-based HPV genotyping and Ct quantification for 14 hrHPV types; (ii) pooling samples with distinct HPV genotypes and similar viral loads; (iii) library preparation, hybrid capture with HPV whole-genome probes, and NGS; and (iv) post-sequencing deconvolution using genotype identity to decode individual sample results. We further developed an automated R/Shiny application to support this workflow, enabling (i) Ct-based pooling optimization, (ii) deconvolution of pooled sequencing results, and (iii) match classification. The visual pipeline is presented in Fig. 2B.

### Application of HPVPool-Seq in clinical samples

A total of 175 HPV-positive cervical exfoliated cell specimens were collected and analyzed using the HPVPool-Seq strategy. All samples were pre-confirmed to be HPV-positive via fluorescent quantitative PCR (q-PCR), covering a wide range of genotypes and viral loads. Based on the qPCR-derived genotype composition and Ct values, pooling was performed with two to four samples per pool (Fig. 1A), ensuring minimal genotype overlap and approximate viral copy number balance. Pooling, library construction, and sequencing were successfully completed for all samples. The majority of pools contained two or three samples (*n* = 33 and *n* = 24 respectively), while only four pools contained four samples. Pooling, library construction, and sequencing were successfully completed for all samples. Pooling size was limited to two to four samples primarily to maintain genotype separation, balance viral load across samples, and accommodate batch-specific constraints.

Among the 175 sequenced samples passing quality control, a total of 45 unique HPV genotypes were detected. These included 14 high-risk types (HPV16, 18, 31, 33, 35, 39, 45, 51, 52, 56, 58, 59, 66, and 68) and 31 low-risk or undetermined-risk types (HPV6, 11, 26, 30, 32, 34, 40, 42, 43, 44, 53, 54, 55, 61, 62, 67, 70, 71, 73, 74, 81, 82, 83, 84, 86, 87, 89, 90, 91, and 101) as illustrated in Fig. S1B. Among high-risk types, HPV16, HPV52, and HPV58 were the most frequently detected, consistent with their known prevalence in the target population. To further examine the integration propensity of each genotype, we compared the number of samples in which each high-risk HPV type was detected to those in which the same type showed evidence of host genome integration (Fig. 2C). The distribution of integration events closely paralleled that of genotype detection, with HPV16, 52, and 58 being predominant in both contexts.

The qPCR-derived Ct values exhibited a wide dynamic range (median Ct = 21.7, interquartile range: 22.9–34.1), indicating inclusion of both high-copy and low-copy samples. The distribution of Ct values, shown in Fig. S1C, demonstrated a bimodal pattern, reflecting heterogeneity in viral load among clinical samples.

### Sequencing overview

High-throughput sequencing generated a total of 164 usable sample libraries. The average raw sequencing data per sample was 5.35 gigabases (Gb), with an average of 5.02 Gb clean data after adapter trimming and quality filtering. The average aligned read data were 2.52 Gb, corresponding to an alignment rate of 52.15%. Duplication rates were generally low, with a mean duplication rate of 10.75% (range: 0.4%–31.6%), suggesting limited PCR amplification artifacts. A summary of sequencing quality metrics is presented in Table S1, including per-sample minimum and maximum aligned data, alignment rates, and duplication statistics.

### Concordance between HPVPool-Seq and qPCR genotyping

**Fig 3:**
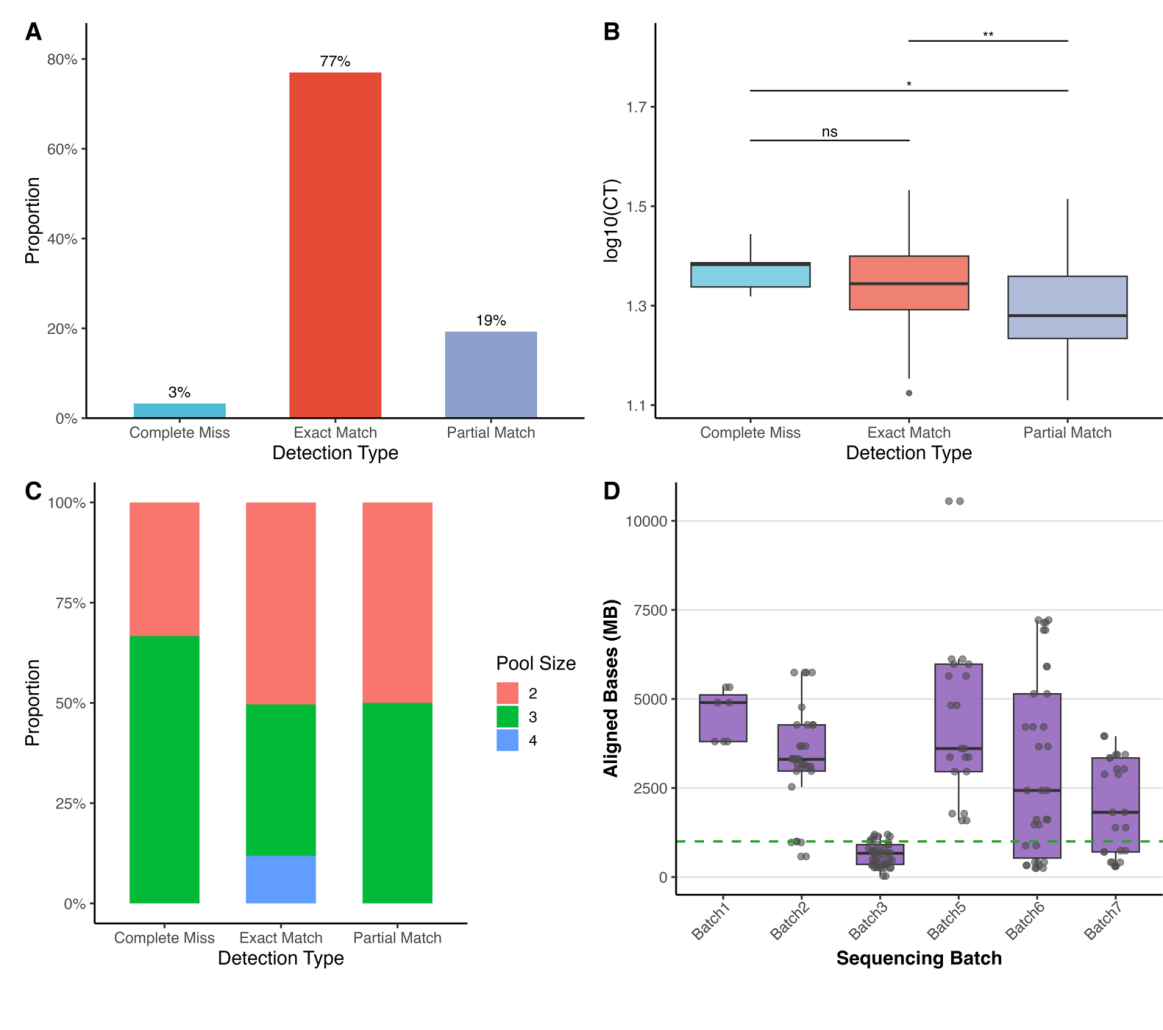
Analysis of concordance and influencing factors for HPVPool-Seq genotyping results. (**A**) Classification of concordance between HPVPool-Seq and reference q-PCR detection for hrHPV genotypes. “Exact match” indicates complete agreement; “partial miss” indicates one or more qPCR-detected genotypes were missed by NGS; “complete miss” indicates no hrHPV detected by NGS. (**B**) Distribution of log-transformed qPCR Ct values among concordance categories. Significant differences were observed between groups (Bonferroni-adjusted *P* < 0.05), suggesting lower viral loads were associated with increased detection failure. (**C**) Pool size distribution across concordance categories. Stacked bar plots indicate no significant association between number of samples per pool and concordance outcome. (**D**) Sequencing quality comparison by batch. Samples from one batch (Batch 3) exhibited significantly lower aligned bases (horizontal dashed line at 1,000 Mb indicates suboptimal alignment quality), suggesting potential technical bias influencing detection performance.

To evaluate the analytical performance of HPVPool-Seq, we compared NGS-detected hrHPV genotypes with qPCR reference results across 175 pooled samples. Among them, 135 cases (77.1%) showed exact concordance, 34 (19.4%) exhibited partial misses (defined in Methods), and 6 (3.4%) were completely missed, with no over-detections observed (Fig. 3A). Cohen’s Kappa coefficient was 0.696 (*P* < 0.001), indicating moderate to substantial agreement between the two methods and demonstrating that pooling-based NGS can reliably replicate qPCR-based genotyping in most cases. To further illustrate the diagnostic performance of our method, we constructed a binary 2 × 2 contingency table treating each genotype detection event as a separate diagnostic instance (Table S2). Pooling-based NGS achieved a sensitivity of 96.9% and a specificity of 86.7% relative to qPCR results, confirming its high concordance with the reference method.

To further investigate the discordant cases identified in this comparison, 14 pooled samples were selected for single-sample NGS retesting. Among these, nine samples (64.3%) recovered at least one hrHPV genotype previously missed in the pooled assay. Notably, fully missed types, such as HPV16, 33, 39, and 52, were successfully detected in individual testing. In parallel, nine cases (64.3%) also revealed new integration events (e.g., HPV39, 52, 66) that were undetected in the pooled setting (Table S3). These results suggest that technical limitations, especially in lower-quality batches (e.g., Batch 3), may contribute to under-detection, and targeted retesting can effectively rescue critical cases.

### Factors associated with detection discordance

To explore possible contributors to detection errors, we performed statistical analyses on several variables, including viral load (roughly evaluated by Ct value), batch-specific effect, pool size, HPV genotypes, and multiple infection status. Ct values were transformed into log10 viral copy numbers for analysis. As shown in Fig. 3B, samples with partial or complete misses had significantly higher Ct values (indicating lower viral loads) compared with exact matches (Bonferroni-adjusted *P* < 0.01), confirming viral load as a major contributor to detection sensitivity. It is to be noted that five of six completely missed samples originated from the same experimental batch, suggesting batch-specific technical errors, likely during library preparation or hybridization, played a role in failure of detection (Table S3).

We also evaluated whether pool size (i.e., the number of samples combined in each pool) affected concordance. Pool sizes ranged from two to four samples per pool and were similar across concordance groups. The Kruskal-Wallis test revealed no significant difference across groups (χ² = 1.21, df  =  2, *P*  =  0.546) (Fig. 3C). While the small sample sizes within each pool-size subgroup may limit statistical robustness, no adverse impact on genotyping accuracy was detected. Interestingly, all samples with a pool size of 4 showed exact matches with qPCR results, suggesting that larger pools may still maintain reliable detection under optimized conditions. This supports the robustness and scalability of HPVPool-Seq across varying pooling strategies. Further sequencing QC metrics showed that Batch 3 exhibited markedly lower aligned read counts (Fig. 3D), reinforcing the impact of experimental variability on detection reliability.

### Cost-efficiency evaluation

**Fig 4:**
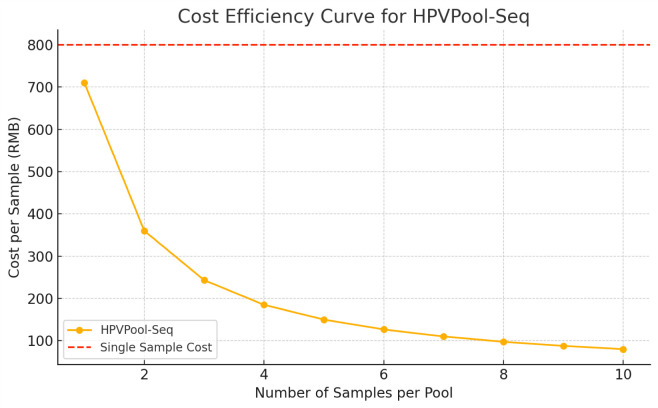
Cost-effectiveness evaluation of the HPVPool-Seq strategy. Total sequencing costs were estimated for processing 1,000 HPV-positive samples using the pooling-based HPVPool-Seq method versus conventional single-sample NGS workflows. The analysis considered variations in pool sizes ranging from two to five samples per pool. While HPVPool-Seq incurs additional qPCR typing and decoding costs, the substantial savings from reduced sequencing library preparation and sequencing runs lead to marked overall cost reductions. The most cost-efficient scenario was observed at a pool size of 4, with up to 60% cost savings compared with the standard approach.

To assess the economic impact of implementing the HPVPool-Seq strategy, we simulated sequencing costs for 1,000 HPV-positive samples under various pooling scenarios. Despite additional costs for qPCR genotyping and computational decoding, the pooling-based approach significantly reduced the number of sequencing libraries and sequencing lanes required. As shown in Fig. 4, pooling three to four samples per library yielded the greatest savings, with up to 60% cost reduction compared to standard single-sample NGS. These results support the potential of HPVPool-Seq to improve cost-efficiency in large-scale HPV genotyping and integration detection applications.

## DISCUSSION

Persistent infection with high-risk HPV and subsequent integration of viral DNA into the host genome are pivotal events in the pathogenesis of cervical cancer and other HPV-associated malignancies (21–23). Detection of HPV integration not only marks a critical transition from transient to transforming infection but also serves as a prognostic biomarker for risk stratification in HPV-positive individuals (10, 24). Although next-generation sequencing (NGS) remains the gold standard for comprehensive integration profiling, its prohibitive cost and technical complexity have limited its application in population-scale screening programs. To overcome this barrier, we developed HPVPool-Seq, a genotype-guided pooling strategy that exploits HPV subtypes as intrinsic biological barcodes to enable cost-efficient targeted sequencing. In a proof-of-concept cohort of 175 HPV-positive clinical specimens, HPVPool-Seq achieved 77.1% exact genotype concordance with qPCR and a 97.1% combined sensitivity when accounting for partial matches, demonstrating strong feasibility for high-throughput integration detection while maintaining diagnostic accuracy.

Recent advances in pooling-based NGS strategies, such as ApharSeq for SARS-CoV-2 detection (25, 25), have shown the potential of early pooling and molecular barcoding to dramatically reduce sequencing costs without sacrificing sensitivity. However, while such approaches have been successful for RNA virus detection, their extension to DNA virus integration analysis, particularly for oncogenic HPV, has not previously been reported. HPVPool-Seq represents the first application of NGS pooling tailored to viral-host integration detection, synergizing pre-screening qPCR data with targeted capture sequencing to achieve both economic efficiency and biological precision. This dual-phase design introduces an important innovation by retaining genotype-level traceability during pooling, offering a self-correcting framework for clinical validation.

Our results further elucidate the robustness and limitations of the HPVPool-Seq approach. A detailed analysis of discordant cases revealed that missed detections were predominantly associated with high Ct values, reflecting lower viral loads, consistent with previous observations that viral copy number critically influences NGS sensitivity. Additionally, batch-specific technical issues were evident, as most (5/6) completely missed cases originated from a single low-quality batch, highlighting the necessity of rigorous process control in high-throughput workflows. Importantly, among 14 discordant pools selected for individual retesting, nine samples (64.3%) successfully recovered at least one previously missed high-risk genotype, and a similar proportion revealed new integration events. These findings validate the built-in rescue capability of HPVPool-Seq. Moreover, the biological complexity of multiplex HPV infections poses intrinsic challenges to detection (26), as illustrated by one case (see Sample ID: 3-3-G8-T9-31 in Table S3) where qPCR identified triple infection (HPV52, 58, and 66), pooled sequencing detected only HPV52, and single-sample retest identified HPV66. Such shifts likely reflect competitive hybridization dynamics and stochastic dropout of low-copy genotypes, phenomena also reported in multiplex pathogen detection studies (27, 28). Interestingly, although the probe set was originally designed to target 32 full-length HPV reference genomes, our sequencing results revealed 45 unique HPV genotypes. This likely reflects a combination of off-target hybridization and potential cross-reactivity due to conserved genomic regions shared among HPV types. While not experimentally validated in this study, such broad-spectrum capture suggests that the hybrid-capture approach may be applicable to broader epidemiological surveillance beyond predefined targets. Further investigation is warranted to clarify the specificity and breadth of probe hybridization across diverse HPV types.

A key strength of HPVPool-Seq lies in its traceable and corrective design. Pooling based on known qPCR genotypes enables real-time identification of inconsistencies, with selective retesting serving as an effective rescue strategy for critical discordant cases. This traceability, coupled with an approximately 60% reduction in per-sample library and sequencing costs, makes HPVPool-Seq a practical and scalable solution for large-scale integration profiling. Furthermore, the development of a shiny-based automated tool for pooling design and result decoding enhances the operational feasibility of the method, facilitating its deployment in population-level studies or molecular epidemiology projects. Importantly, HPVPool-Seq is intended for large-scale epidemiological research and biobank settings rather than direct clinical diagnosis. Its cost-efficiency and scalability make it particularly valuable for public health surveillance, HPV-related cancer prevention, and early triage strategies in resource-limited environments.

Nonetheless, several limitations merit consideration. Pooling inherently reduces per-sample sequencing depth, which may limit sensitivity for very low-copy infections, especially in cases of coexisting low-abundance genotypes. Specificity could not be directly assessed in this study because all samples were pre-confirmed as HPV-positive. Although a 77% concordance rate may be suboptimal for diagnostic use, most missed cases had low viral loads or occurred in a single underperforming batch. Our built-in comparison with qPCR enables timely identification of such events. Nevertheless, pooling may not be suitable for targeted clinical testing where maximum sensitivity is required, and individualized testing remains preferred in such contexts.

Additionally, accurate resolution of HPV integration breakpoints remains technically and biologically challenging, especially in early-stage or low-viral load samples. Previous studies have shown that virus-host junctions are often heterogeneous within individual lesions, partly due to structural variations and stochastic read capture during sequencing (8, 13). Our own prior work using long-read sequencing (29) demonstrated that even without error correction, integration breakpoints display micro-variability across technical replicates, suggesting that exact coordinate reproducibility is not always achievable. In our current study, re-sequencing of discordant samples confirmed HPV integration status in most cases but revealed limited overlap in exact breakpoints, likely reflecting both biological diversity and batch-related technical noise. These observations support a growing consensus that integration status, rather than precise breakpoint coordinates, may be more informative for clinical risk stratification.

In conclusion, HPVPool-Seq offers a novel, cost-effective, and accurate strategy for high-throughput HPV integration detection. By creatively leveraging biological barcoding inherent to HPV genotypes, this method bridges the gap between high-resolution molecular profiling and economic feasibility. Our findings support the integration of pooling-based NGS approaches into large-scale HPV molecular epidemiology and screening programs, paving the way for more accessible and scalable precision prevention strategies against HPV-related malignancies.

## Data Availability

The data supporting the findings of this study are available from the corresponding author upon reasonable request.

## SUPPLEMENTAL MATERIAL

Supplemental Material spectrum.01399-25-s0001.pdf Figures S1 to S3; Tables S1 to S3.

ASM does not own the copyrights to Supplemental Material that may be linked to, or accessed through, an article. The authors have granted ASM a non-exclusive, world-wide license to publish the Supplemental Material files. Please contact the corresponding author directly for reuse.
