# Supplementary material for: HPVPool-Seq: a genotype-guided pooling strategy for cost-effective next-generation sequencing detection of HPV integration in cervical samples
Source: Microbiol Spectr. 2025 Aug 19;13(10):e01399-25. doi: 10.1128/spectrum.01399-25 (PMC12502606; doi:10.1128/spectrum.01399-25)
Supplement: Supplemental Material — Figures S1 to S3; Tables S1 to S3. [file spectrum.01399-25-s0001.pdf]

Supplementary data

For

HPVPool-Seq: A Genotype-Guided Pooling Strategy for Cost-Effective Next-Generation Sequencing Detection of HPV Integration in Cervical Samples

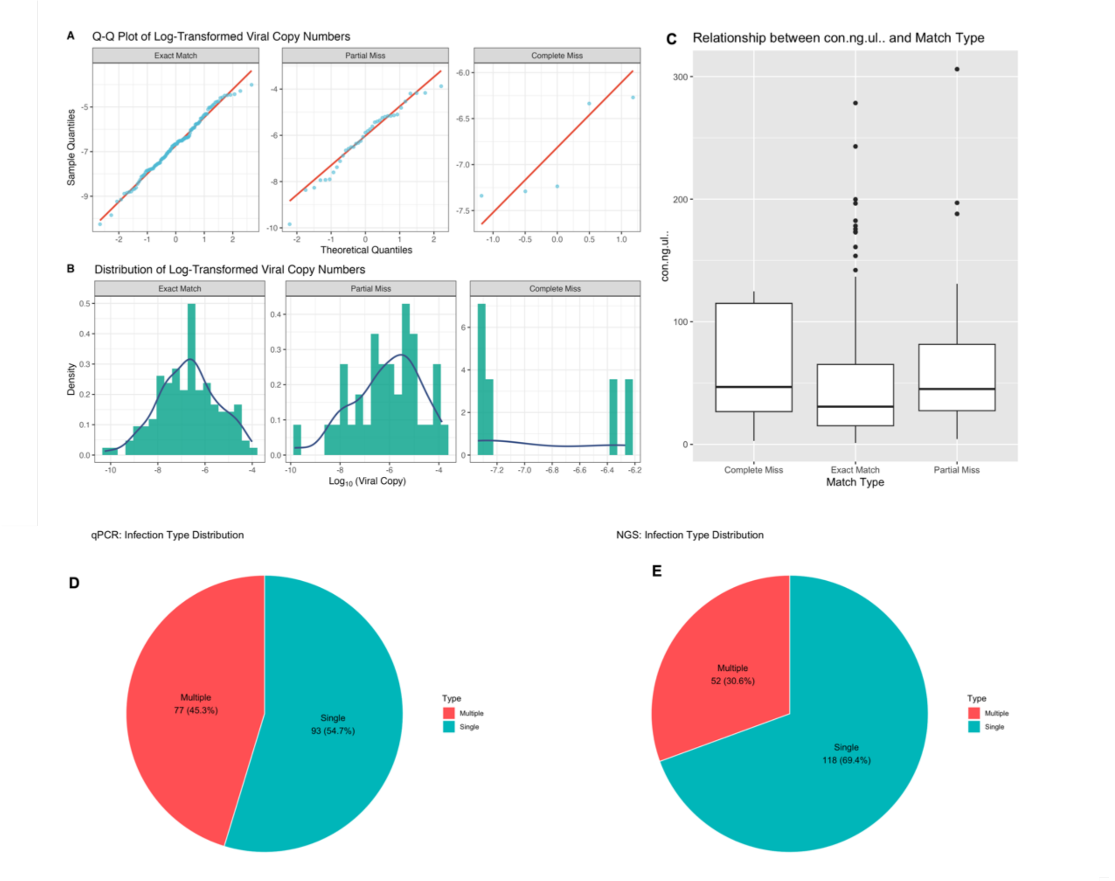

Figure S1. Profiling of Pool Size, Viral Load, and HPV Genotype Spectrum in the HPVPool-Seq Dataset. (A) Q-Q plots of log-transformed Ct values (log10(1/2Ct)) stratified by detection concordance category, indicating near-normal distribution within each group. (B) Density plots of log-transformed Ct values further demonstrate the distributional shift toward higher Ct (lower viral load) among discordant samples. (C) Scatter plot of total DNA concentration (Qubit ng/μL) versus concordance classification, showing no apparent association between input DNA quantity and genotyping agreement. (D–E) Proportion of multiple hrHPV infections based on qPCR and NGS, respectively. Although qPCR detected a higher rate of multiple infections, no significant association was found between multiple infection status and detection discordance in our cohort.

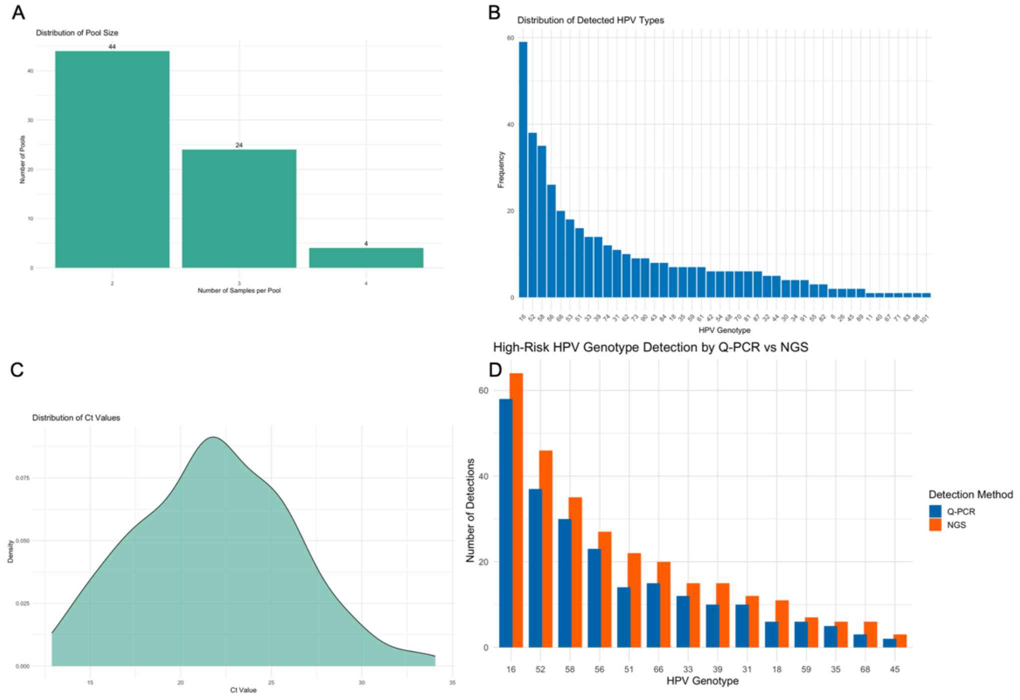

Figure S2. Supplementary analysis of factors associated with HPVPool-Seq detection concordance. (A) Distribution of pooling sizes across all analyzed samples. Each bar represents the number of pools containing a specific number of samples, demonstrating the variability in pool composition (range: 2-4 samples per pool). (B) Overall distribution of HPV genotypes detected by NGS. All 45 identified types are shown, including both high-risk and low-risk genotypes. (C) Density plot of Ct values from qPCR assays for all included samples. The distribution reveals broad variation in viral loads across the cohort, with a bimodal tendency indicating both high and low copy-number populations. (D) Comparison of hrHPV genotype detection frequencies between qPCR-based typing (gray bars) and NGS-based pooling detection (red bars). Only the 14 clinically recognized high-risk types are included. NGS and qPCR show consistent detection patterns for dominant types such as HPV16, 52, and 58, while discordance in minor genotypes may reflect dilutional or capture variability.

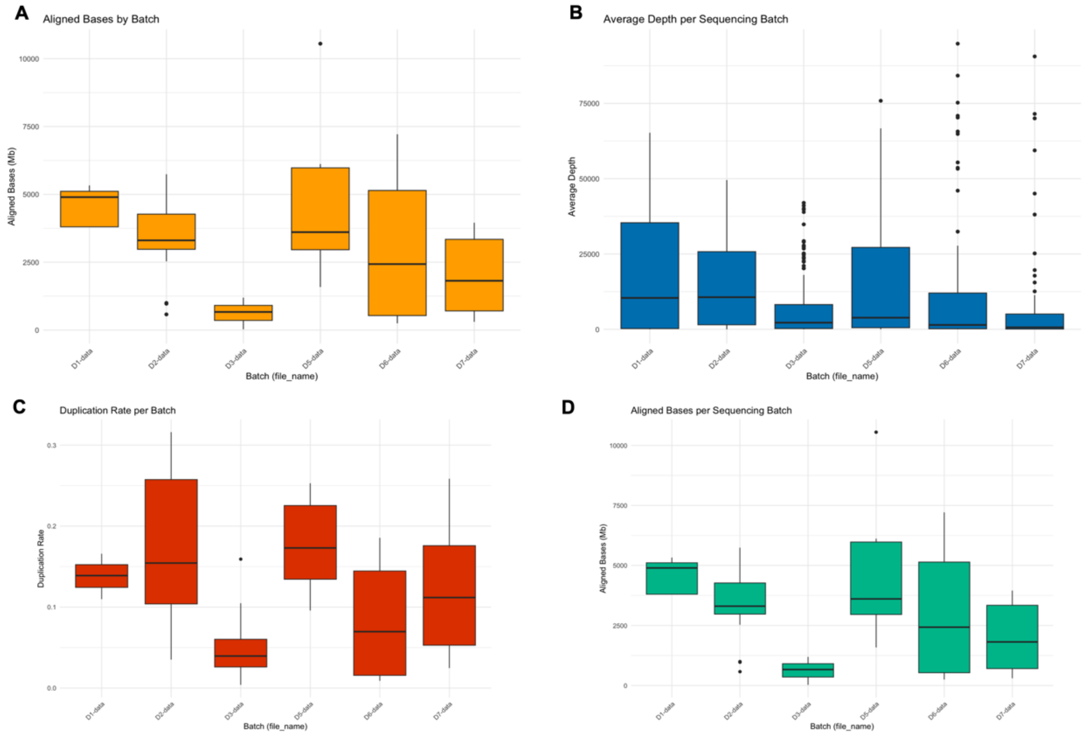

Figure S3. Sequencing quality control metrics across different batches. (A–D) Comparison of four key sequencing quality control indicators among sequencing batches: (A) Raw data yield (Mb), (B) Clean data yield (Mb), (C) Aligned data (Mb), and (D) Alignment rate (%). Batch 3 exhibited significantly lower values across all metrics, supporting the hypothesis that technical failures in this batch contributed to complete detection failures in certain samples.

Table S1 Summary of sequencing quality metrics for all samples.

**file_name**	**pooling_ID**	**Sample**	**Raw_data_bases.Mb.**	**Clean_data_bases.Mb.**	**Aligned_bases.Mb.**	**Aligned**	**Duplication**
D1-data	G1(1,9)	22C519087_R302_CapNGS	8515.89	7357.22	5329.04	0.7243	0.1659
D1-data	G2(5,11,13)	22C519088_R302_CapNGS	7896.87	6710.02	4897.2	0.7298	0.1098
D1-data	G3(2,8,14)	22C519089_R302_CapNGS	8539.12	7298.74	3803.62	0.5211	0.1389
D2-data	2-G1	22C561102_R302_CapNGS	4303.74	3951.6	577.29	0.1461	0.0496
D2-data	2-G2	22C561103_R302_CapNGS	4894.97	4489.31	997.84	0.2223	0.0514
D2-data	2-G3	22C561104_R302_CapNGS	6056.94	5463.26	4271.64	0.7819	0.2575
D2-data	2-G4	22C561105_R302_CapNGS	5109.85	4665.55	3321.9	0.712	0.1544

D2-data	2-G5	22C561106_R302_CapNGS	5119.87	4627.71	3675	0.7941	0.1915
D2-data	2-G6	22C561107_R302_CapNGS	4954.82	4519.89	3096.13	0.685	0.1297
D2-data	2-G7	22C561108_R302_CapNGS	4370.92	3983.49	2530.91	0.6354	0.2036
D2-data	2-G8	22C561109_R302_CapNGS	6033.75	5536.33	4768.52	0.8613	0.316
D2-data	2-G9	22C561110_R302_CapNGS	4556.11	4164.32	3304.47	0.7935	0.1432
D2-data	2-G10	22C561111_R302_CapNGS	6189.79	5641.72	3155.75	0.5594	0.1039
D2-data	2-G11	22C561112_R302_CapNGS	4633.64	4259.62	970.3	0.2278	0.0352
D2-data	2-G12	22C561113_R302_CapNGS	5076.87	4659.31	2974.99	0.6385	0.166
D2-data	2-G13	22C561114_R302_CapNGS	6908.61	6288.95	5742.03	0.913	0.282
D3-data	3-3-G8	23C651223_R302_CapNGS	1249.65	1130.41	910.04	0.8051	0.1592
D3-data	3-3-G9	23C651224_R302_CapNGS	1339.14	1209.38	671.59	0.5553	0.0864
D3-data	3-3-G6	23C651225_R302_CapNGS	1210.01	1121.39	666.59	0.5944	0.0548
D3-data	3-3-G3	23C651226_R302_CapNGS	1147.34	1058.36	682.76	0.6451	0.027
D3-data	3-3-G5	23C651227_R302_CapNGS	1604.69	1455.81	1140.4	0.7833	0.0857
D3-data	3-3-G7	23C651228_R302_CapNGS	1064.18	986.08	620.9	0.6297	0.1048
D3-data	3-3-G2	23C651229_R302_CapNGS	1299.33	1196.33	713.24	0.5962	0.0172
D3-data	3-3-G1	23C651230_R302_CapNGS	1183.95	1072.16	909.58	0.8484	0.0461
D3-data	3-3-G4	23C651231_R302_CapNGS	1191.96	1094.06	491.87	0.4496	0.0396
D3-data	3-4-G4	23C651232_R302_CapNGS	1077.61	1001.78	246.15	0.2457	0.004
D3-data	3-4-G6	23C651233_R302_CapNGS	1093.32	1019.47	330.88	0.3246	0.0148
D3-data	3-4-G1	23C651234_R302_CapNGS	1162.48	1069.32	472.53	0.4419	0.0603
D3-data	3-5-G2	23C651235_R302_CapNGS	1117	1022.36	355.09	0.3473	0.0407

D3-data	3-5-G5	23C651236_R302_CapNGS	945.65	886.82	28.11	0.0317	0.0059
D3-data	3-5-G1	23C651237_R302_CapNGS	757.57	711.73	272.57	0.383	0.0261
D3-data	3-5-G4	23C651238_R302_CapNGS	1737.7	1581.02	1192.92	0.7545	0.0715
D3-data	3-5-G12	23C651239_R302_CapNGS	1215.58	1132.26	795.95	0.703	0.0572
D3-data	3-5-G13	23C651240_R302_CapNGS	1238.49	1149.78	988.46	0.8597	0.0329
D3-data	3-5-G7	23C651241_R302_CapNGS	1161.27	1083.5	265.45	0.245	0.0372
D3-data	3-5-G11	23C651242_R302_CapNGS	1305.57	1189.57	1046.75	0.8799	0.094
D3-data	3-5-G8	23C651243_R302_CapNGS	1444.86	1331.44	741.54	0.5569	0.029
D5-data	5-G5	23C740610_R302_CapNGS	5613.6	5110.95	2959.72	0.5791	0.1591
D5-data	5-G4	23C740609_R302_CapNGS	6408.44	5898.99	4821.83	0.8174	0.1344
D5-data	5-G1	23C740606_R302_CapNGS	5723.9	5310.8	3366.35	0.6339	0.173
D5-data	5-G8	23C740613_R302_CapNGS	5100.27	4643.95	1780.69	0.3834	0.1511
D5-data	5-G6	23C740611_R302_CapNGS	5254.96	4814.68	3607.13	0.7492	0.1872
D5-data	5-G7	23C740612_R302_CapNGS	8058.38	7449.81	5644.91	0.7577	0.2256
D5-data	5-G2	23C740607_R302_CapNGS	12619.42	11228.29	6117.45	0.5448	0.2254
D5-data	5-G9	23C740614_R302_CapNGS	6874.09	6502.97	1584.91	0.2437	0.0958
D5-data	5-G10	23C740615_R302_CapNGS	7762.47	7137.81	5976.36	0.8373	0.1089
D5-data	5-G3	23C740608_R302_CapNGS	18825.7	17553.54	10553.18	0.6012	0.2529
D6-data	6-G13	24C019003_R302_CapNGS	9458.3	9113.66	5143.21	0.5643	0.1579
D6-data	6-G9	24C018999_R302_CapNGS	10145.89	9897.87	881.92	0.0891	0.0251
D6-data	6-G11	24C019001_R302_CapNGS	8725.94	8429.34	3662.8	0.4345	0.0697
D6-data	6-G12	24C019002_R302_CapNGS	11235.78	10875.82	5909.39	0.5434	0.1047

D6-data	6-G4	24C018995_R302_CapNGS	8492.55	8222.99	327.3	0.0398	0.0135
D6-data	6-G1	24C018992_R302_CapNGS	8817.5	8446.74	4213.96	0.4989	0.1856
D6-data	6-G7	24C018998_R302_CapNGS	8959.17	8637.42	2430.35	0.2814	0.0714
D6-data	6-G14	24C019004_R302_CapNGS	9663.88	9292.71	6932.95	0.7461	0.1621
D6-data	6-G10	24C019000_R302_CapNGS	9700.17	9379.23	7145.79	0.7619	0.1016
D6-data	6-G2	24C018993_R302_CapNGS	9334.1	9093.45	417.22	0.0459	0.0129
D6-data	6-G5	24C018996_R302_CapNGS	10173.48	9895.3	1613.77	0.1631	0.0311
D6-data	6-G15	24C019005_R302_CapNGS	9670.43	9349.62	7214.09	0.7716	0.1799
D6-data	6-G6	24C018997_R302_CapNGS	9830.16	9590.85	1471.02	0.1534	0.0223
D6-data	6-G3	24C018994_R302_CapNGS	9512.19	9219.27	249.66	0.0271	0.009
D7-data	7-G1	24C136280_R302_CapNGS	3955.4	3864.88	302.28	0.0782	0.0247
D7-data	7-G2	24C136281_R302_CapNGS	3349.55	3292.1	414.16	0.1258	0.0529
D7-data	7-G3	24C136282_R302_CapNGS	3743.88	3685	739.37	0.2006	0.0635
D7-data	7-G4	24C136283_R302_CapNGS	3794.59	3726.45	1816.12	0.4874	0.1759
D7-data	7-G5	24C136284_R302_CapNGS	4623.95	4494	1388.89	0.3091	0.0354
D7-data	7-G6	24C136285_R302_CapNGS	4068.91	4004.53	2883.23	0.72	0.2585
D7-data	7-G7	24C136286_R302_CapNGS	4131.03	4047.41	3435.66	0.8489	0.1118
D7-data	7-G8	24C136287_R302_CapNGS	4631.27	4532.12	3955.18	0.8727	0.1886
D7-data	7-G9	24C136288_R302_CapNGS	4840.8	4689.69	3343	0.7128	0.1732
D7-data	7-G10	24C136289_R302_CapNGS	3102.94	3063.58	704.64	0.23	0.1094
D7-data	7-G11	24C136290_R302_CapNGS	3382.17	3310.55	3032.66	0.9161	0.2546

Table S2 A 2×2 contingency table comparing genotype-level detection results from qPCR and pooling-based NGS.

	**NGS Positive**	**NGS Negative**	**Total**
qPCR Positive (True Positive + False Negative)	222 (TP)	7 (FN)	229
qPCR Negative (False Positive + True Negative)	34 (FP)	222 (TN)	256
**Total**	**256**	**229**	**485**

Sensitivity: 222 / (222 + 7) ≈ 96.9%; Specificity: 222 / (222 + 34) ≈ 86.7%. Each genotype detection event per sample is treated as a separate binary test. All included samples were known to be HPV-positive by clinical screening, hence the dataset was enriched for true positives. The table demonstrates the ability of our pooling-based method to sensitively and specifically replicate the reference qPCR genotyping.

Table S3 Detailed results of 14 pooled samples selected for single-sample NGS retesting.

Seg Batch	Sample_ID	qPCR_Genotype	NGS.hpv	NGS.retest	Recovered_Genotype	NGS.intergration	NGSinter.retest	Recovered_Integration
3	3-3-G7-T9-35	16	N	16	16	N	16	16
3	3-3-G9-T9-42	16;52	N	16	16	N	16	16
3	3-5-G4-T9-28	33;52	52	52;33	33	52	33;52	33
3	3-3-G7-T9-41	33	N	33;66;68;51	33	N	33;66;68;51	33;66;68;51
1	1-G2-T3-5	39;58;68	58	58;39	39	58	39;58	39
2	2-G8-T5-23	56;66;52;45	52;56;66	52;66;56;53;45	45	66	52;53;56;66	52;53
3	3-3-G6-T9-43	16;52	16	52	52	16	52	52
3	3-5-G7-T9-26	16;58	16	16;58	58	N	16;58	16;58
3	3-3-	58;66;5	52	66	66	N	66	66

	G8-T9-31	2						
3	3-5-G3-T9-24	16;33;51;39	N	16;39;51	16;39;51	N	16;39;51	16;39;51
3	3-5-G7-T9-30	52;39	N	52;39	52;39	N	52;39	52;39
2	2-G7-T5-24	51;39	51	51;53	None	51	51;53	None
3	3-3-G1-T6-2	16;51;45;39	39;45;51	39;45;51	None	45;51;39	39;45;51	None
2	2-G8-T5-22	52;59;39;51	39;52	39;52	None	39	39	None
